# The Critical Shoulder Angle Can be Accurately and Reliably Determined from Three‐Dimensional Computed Tomography Images

**DOI:** 10.1111/os.13652

**Published:** 2023-01-20

**Authors:** Yi Long, Huijun Hu, Chuanhai Zhou, Jingyi Hou, Zhiling Wang, Min Zhou, Dedong Cui, Xiaoding Xu, Rui Yang

**Affiliations:** ^1^ Department of Orthopaedics, Sun Yat‐sen Memorial Hospital Sun Yat‐sen University Guangzhou China; ^2^ Department of Radiology, Sun Yat‐sen Memorial Hospital Sun Yat‐sen University Guangzhou China; ^3^ Guangdong Provincial Key Laboratory of Malignant Tumor Epigenetics and Gene Regulation, Sun Yat‐sen Memorial Hospital Sun Yat‐sen University Guangzhou China

**Keywords:** Antero‐posterior radiographs, Critical shoulder angle, Rotator cuff tears, Three‐dimensional computed tomography

## Abstract

**Objective:**

Anteroposterior (AP) radiographs do not necessarily offer the optimal approach to measuring the critical shoulder angle (CSA) due to the malposition of the scapula. Three‐dimensional computed tomography (3D‐CT) may offer some advantages, including the ability to rotate the scapula for position alignment and pre‐operative planning for reducing CSA. This study aimed to investigate the accuracy and reliability of CSA measurement in 3D‐CT and to determine whether there is an association between CSA and rotator cuff tears (RCTs).

**Methods:**

In this retrospective study we identified 200 patients who received shoulder arthroscopy from 2019 to 2021, including 142 patients (81 females, 61 males) with RCTs and 58 patients (14 females, 44 males) with non‐RCTs. For each participant, CSA was measured from standard shoulder AP radiographs and anterior views of 3D‐CT of the scapula by two independent assessors. Inter‐ and intra‐observer agreements were assessed by the intraclass correlation coefficient (ICC). The relationship between the two measurement methodologies was determined by Spearman's correlation coefficient and Bland–Altman plots. Discriminative capacity was calculated by using receiver operating curve (ROC) analyses in the whole cohort and age sub‐groups above and below 45 years.

**Results:**

We found perfect inter‐observer (ICC >0.96) and intra‐observer (ICC >0.97) reliabilities for CSA measurements obtained from the standard AP radiographs and the 3D‐CT. There was a strong correlation between the two methods (*r* = 0.960, *P* < 0.001). The mean CSA was 31.7° ± 4.2° in the standard AP radiographs and 31.8° ± 4.4° in the 3D‐CT (mean difference 0.02°, *P* = 0.940; bias 0.02°, limits of agreement −2.29° to +2.33°). ROC analysis of the whole cohort showed that the CSA measured in the standard AP radiographs (area under the ROC curve [AUC] = 0.812, *P* < 0.001) and the 3D‐CT (AUC = 0.815, *P* < 0.001) predicted RCT with high confidence. ROC analysis of patients aged ≥45 years showed that the CSA measured from the standard AP radiographs (AUC = 0.869, *P* < 0.001) and the 3D‐CT (AUC = 0.870, *P* < 0.001) were very good at predicting RCTs.

**Conclusion:**

CSA measured from standard AP radiographs and 3D‐CT showed high consistency, and the CSA could be accurately and reliably measured using 3D‐CT. CSAs measured from standard AP radiographs and 3D‐CT could predict RCTs, especially in patients aged ≥45 years.

## Graphical abstract

Critical shoulder angle (CSA) measured in the standard anteroposterior radiographs and 3D‐CT manifest a high consistency. CSA could be accurately and reliably measured in the 3D‐CT, which would be potentially advantageous in pre‐operative planning for acromioplasty.

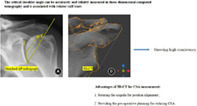

## Introduction

The critical shoulder angle (CSA) has been demonstrated to be an effective predictor for the development of shoulder pathology.^1^, ^2^, ^3^, ^4^ Numerous studies have documented that CSA values >33–35° are correlated with rotator cuff tears (RCTs) and a higher retear rate after arthroscopic rotator cuff repair (RCR). CSAs <30° are associated with glenohumeral osteoarthritis (GHOA).^5^, ^6^, ^7^, ^8^, ^9^, ^10^, ^11^, ^12^ Hence, accurate measurement of CSA and reduction of excessive CSA to a desirable range by acromioplasty is expected to offer marked clinical benefit.

However, the CSA involves a two‐dimensional radiographic measurement of a three‐dimensional structure. As such. the accuracy of the CSA measurement is dependent on the quality of the anteroposterior (AP) radiograph.^1^, ^13^ Suter *et al*. quantified the influence of the scapular position on CSA measurement deviation and proposed the Suter–Henninger (SH) scapular classification system, which re‐defined the standard AP radiographs (A1 and C1) to measure the CSA accurately. CSA measured on a non‐standard AP radiograph is considered to be unreliable.^14^ Unfortunately, high‐quality standard AP radiographs that meet SH criteria are difficult to obtain. Several published articles have reported that over 70% of shoulder films fail to meet the requirements for standard AP radiographs.^5^, ^15^, ^16^

Theoretically, in contrast to plain radiography, three‐dimensional computed tomography (3D‐CT) shows greater potential for accurate measurement of the CSA since the standardized anterior view can be established by rotating the scapula by utilizing its bony markers. Additionally, accurate pre‐operative planning to reduce the CSA can be performed by utilizing 3D‐CT. Several research teams have performed pre‐operative planning for acromioplasty to accurately reduce excessive CSA (CSA >35°) to the desired range (30–33°).^17^, ^18^, ^19^, ^20^, ^21^, ^22^ However, there is little evidence supporting the accuracy and reliability of CSA measurements from 3D‐CT, which impairs clinical decision‐making.

In this study, we aimed to investigate the accuracy and reliability of CSA measurements from the anterior 3D‐CT view compared to standard AP radiographs. We also aimed to determine whether there is an association between the CSA and RCTs. We hypothesized that 3D‐CT would offer an accurate and reliable method to measure the CSA, and that an association exists between the CSA and RCTs.

## Methods

### Patient Selection

This study was approved by the ethics committee of Sun Yat‐sen Memorial Hospital (SYSEC‐KY‐KS‐2018‐036). Between 2019 and 2021, all consecutive patients who received arthroscopic treatment of the shoulder in the department of orthopedics were screened retrospectively. Inclusion criteria included: (i) simultaneous pre‐operative shoulder CT scans and AP radiographs of the affected shoulder at our hospital; and (ii) aged 18 years or older. Exclusion criteria included: (i) quality of the AP radiographs does not meet the type A1 and C1 of SH criteria;^14^ (ii) CT scans do not include the entire scapula; (iii) scapula fracture or tumor; and (iv) moderate to severe defects of the glenoid, including bony Bankart lesions or defects resulting in the inability to measure CSA accurately. For the RCTs group, patients with isolated subscapularis tears were excluded as the CSA was considered independent of subscapularis injury.^23^, ^24^ All patients were diagnosed based on a pre‐operative physical examination combined with imaging findings and finally confirmed by shoulder arthroscopy.

### Radiographic Assessment

The Digital Imaging and Communications in Medicine (DICOM) data of shoulders were obtained from the department of radiology. Then, the DICOM data were imported into Mimics 20.0 (Materialise, Leuven, Belgium) for 3D reconstructions. Next, Blender 2.81 software (Amsterdam, the Netherlands) was used for 3D shoulder model position alignment according to methods published in previous studies.^25^, ^26^

**Fig. 1:**
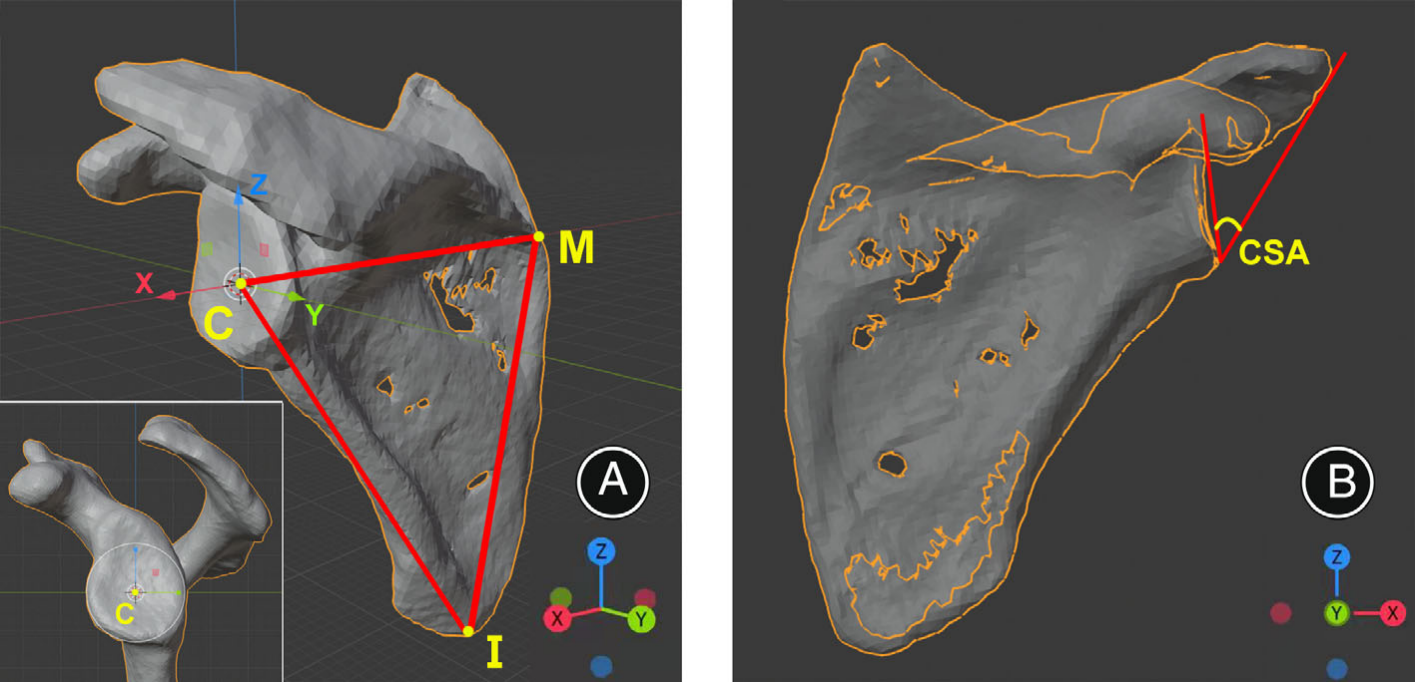
Coordinate system establishment on the 3D shoulder model (left shoulder). (A) The scapular plane (*I‐C‐M*) was identified by three points at the best‐fit circle center of the glenoid (*C*), the inferior scapular angle (*I*), and the point where the scapular spine intersects the medial border of the scapula (*M*). The x‐axis connecting the points *M* and *C*, y‐axis normal to the scapular plane, and the z‐axis is defined by the cross product of x‐ and y‐axes. (B) The anterior 3D‐CT view of the scapula was defined as perpendicular to the scapula plane along the y‐axis, and the scapula was toggled to the X‐ray model in this view for CSA measurement.

The Cartesian coordinate system was established so that every 3D shoulder model position would be aligned to a standardized reference system. The main procedures of position alignment were as follows. First, the scapular plane of each 3D model was identified; defined by three anatomical landmarks: the inferior scapular angle (*I*), the point where the scapular spine intersects the medial border of the scapula (*M*), and the best‐fit circle center of the glenoid (*C*), which was the center of the inferior part of the glenoid.^25^, ^27^ Next, the x‐axis was defined by the line *M‐C*, the y‐axis was perpendicular to the scapular plane (*C‐I‐M* plane), and the z‐axis was defined by the cross product of the x‐ and y‐axes. Consequently, the Cartesian coordinate system was generated with the x‐, y‐, and z‐axes (Fig. Fig. 1A). The anterior view of 3D‐CT of the scapula was defined as perpendicular to the scapula plane (*C‐I‐M* plane) along the y‐axis. Eventually, the scapula was toggled to the X‐ray model in this view for CSA measurement (Fig. Fig. 1B).

**Fig. 2:**
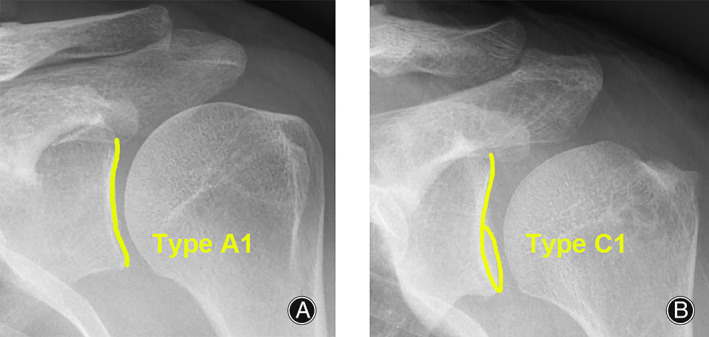
Radiographic films detailing type A1 and type C1 according to the Suter‐Henninger (SH) scapular classification system (left shoulders). (A) The type A1 film was defined as the anterior and posterior glenoid rims completely overlapping, and with overlap of the superior glenoid border and the coracoid process. (B) The type C1 film was defined as the anterior and posterior glenoid rims partially overlapping in the inferior 50% of the glenoid, and with overlap of the superior glenoid border and the coracoid process.

**Fig. 3:**
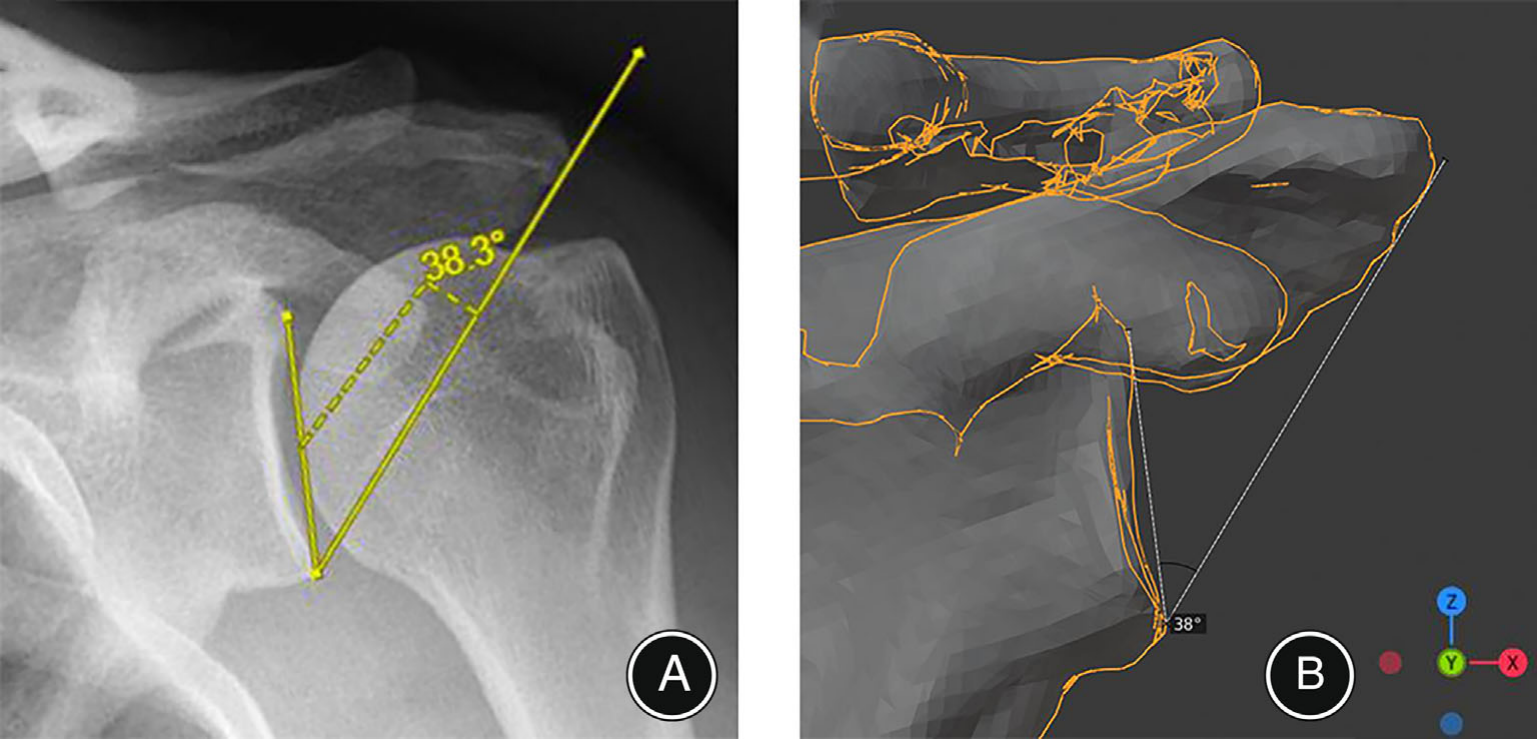
The measurement of critical shoulder angle (CSA) for the same patient (left shoulder). (A) Standard anteroposterior radiograph showed that the CSA was 38.3°. (B) The anterior view of 3D‐CT showed CSA was 38°.

According to the SH criteria, the type A1 of the AP radiographs was defined as the anterior and posterior glenoid rims completely overlapping, and there was an overlap of the superior glenoid border and the coracoid process (Fig. 2A). The type C1 of the AP radiographs was defined as the anterior and posterior glenoid rims partially overlapping in the inferior 50% of the glenoid, and there was overlap of the superior glenoid border and the coracoid process (Fig. 2B). The type A1 and C1 radiographs were regarded to reflect standard AP radiographs.^14^ The CSA was measured from standard AP radiographs (Fig. 3A) and anterior 3D‐CT views of the scapula (Fig. 3B) for each included patient. The CSA was defined as the angle between the line connecting the superior and inferior borders of the glenoid and the line connecting the inferior border of the glenoid with the most inferolateral point of the acromion.^1^

### Statistical Analysis

For *a priori* power analysis, we calculated the sample size using PASS 15 (NCSS LLC, Kaysville, UT, USA) by utilizing data reported by Incesoy *et al*.^2^ The mean CSA for the RCTs group was 33.6° ± 3.9° and 31.5° ± 4.0° for the non‐RCTs group. We assumed a 3:1 ratio of RCTs to non‐RCTs patients, based on the expected difficulty of locating non‐RCTs individuals and the expected size of the RCTs group. Thus, for a power of 0.85 and an alpha error of 0.05, we determined that we required a sample size of 43 patients in the non‐RCTs group and 129 patients in the RCTs group.

The CSA was measured by two independent assessors (one orthopedist and one radiologist, all with more than 5 years of experience); this was repeated by the orthopedist after 1 month. Interclass correlation coefficients (ICC) were assessed by examining inter‐ and intra‐observer agreement. We considered ICCs of 0.75 or higher to be sufficient for reliability.^28^ Data were assessed for normality with the Shapiro–Wilk test. Independent sample *t*‐tests (normally distributed data) and Mann–Whitney *U*‐tests (non‐normally distributed data) were used to compare groups. The chi‐square test was used to compare qualitative data. Spearman's correlation coefficients were calculated to evaluate the relationship between the CSA measured in the standard AP radiographs and 3D‐CT. Inter‐method comparisons were performed using the Wilcoxon signed rank test and Bland–Altman plots. Receiver operating curves (ROC) were generated for the CSAs in the whole cohort and age sub‐groups above and below 45 years. An area under the ROC curve (AUC) 0.70–0.80 was taken to indicate good discrimination, and an AUC >0.80 to indicate excellent discrimination.^13^ Summary data of continuous variables were shown as mean and standard deviation (mean ± SD). The statistical significance was set at *P* < 0.05. All statistical analyses were performed using SPSS 22 (IBM, Armonk, NY, USA) and MedCalc 19.0 (MedCalc, Ostend, Belgium).

## Results

### General Results

**Fig. 4:**
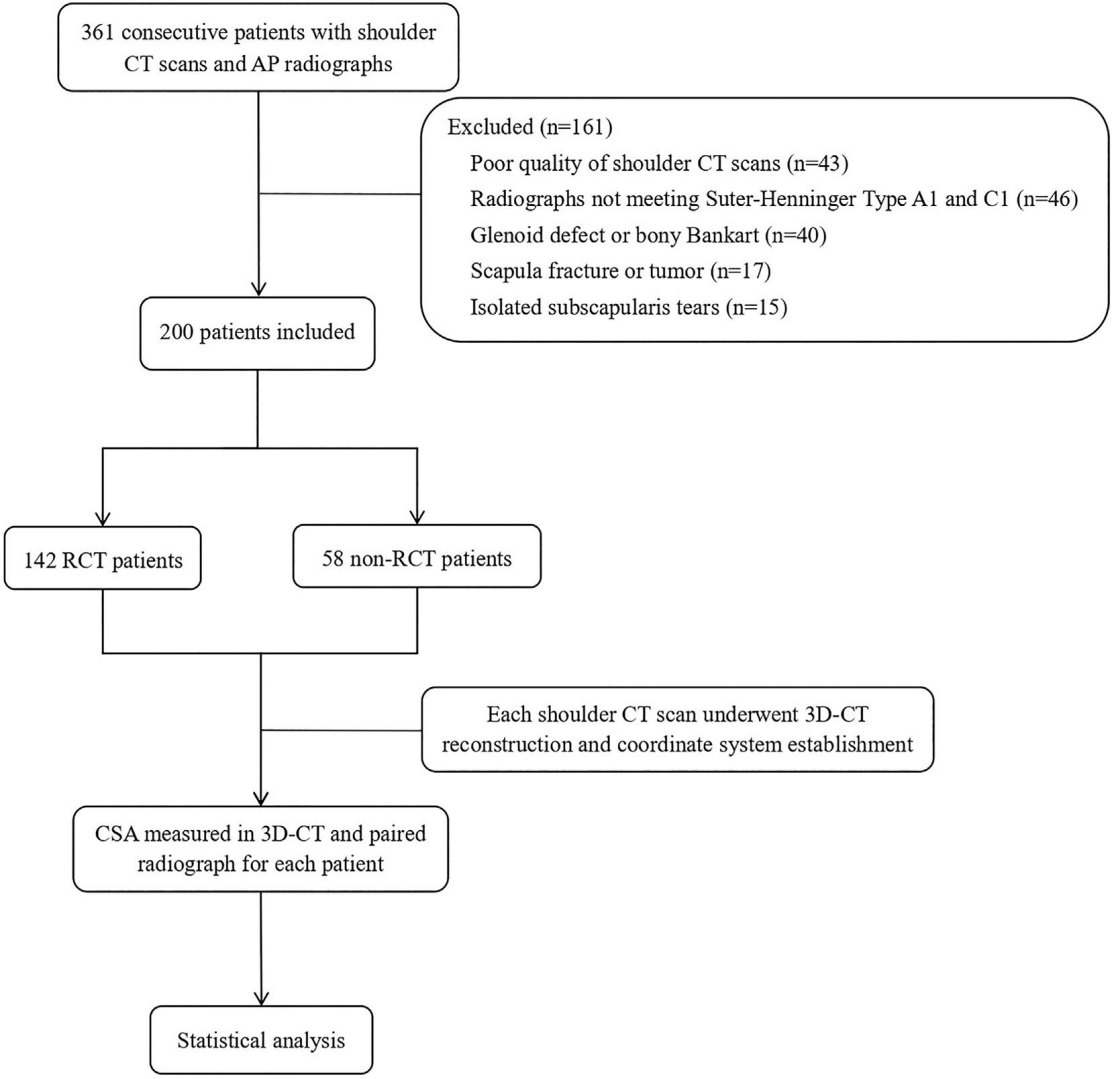
Flowchart of the progress through the study phases.

Initially, 361 patients met our inclusion criteria. Of these, 43 patients were excluded due to poor CT scan quality, 46 due to non‐standard AP radiographs, 40 due to glenoid defects or bony Bankart lesions, 17 due to scapula fractures or tumors, and 15 due to isolated subscapularis tears. Ultimately, 200 patients were included in the study. Of these, 142 had RCTs, and 58 had intact rotator cuffs but with shoulder dislocations, SLAP lesions, and other associated abnormalities (Fig. 4). Patient characteristics are summarized in Table 1.

**TABLE 1:** Demographic characteristics of the study participants

Characteristic	RCTs group (*n* = 142)	Non‐RCTs group (*n* = 58)	Statistic value	*P* value
Age, mean ± SD (range)	56.8 ± 12.8 (19–93)	39.0 ± 15.4 (18–72)	*U* = 1640.000	<0.001*
Age group			*χ* ^2^ = 39.067	<0.001*
<45 years	28	38		
≥45 years	114	20		
Sex			*χ* ^2^ = 17.879	<0.001*
Male	61	44		
Female	81	14		
Laterality			*χ* ^2^ = 0.839	0.0360
Left	42	21		
Right	100	37		
CSA, deg				
X‐ray, mean ± SD (range)	33.1 ± 3.6 (25.1–41.7)	28.4 ± 3.6 (20.9–36.5)	*U* = 1550.000	<0.001*
3D‐CT, mean ± SD (range)	33.2 ± 3.8 (25.0–41.0)	28.2 ± 3.8 (21.0–36.0)	*U* = 1524.500	<0.001*

Abbreviations: 3D‐CT, three‐dimensional computed tomography; CSA, critical shoulder angle; RCTs, rotator cuff tear; SD, standard deviation.

* Statistically significant.

### Inter‐ and Intra‐Observer Reliability

CSA measurements from the standard AP radiographs and 3D‐CT showed perfect inter‐observer (ICC >0.96) and intra‐observer (ICC >0.97) reliability for CSA measurement. The raw data for the inter‐ and intra‐observer reliability calculations are summarized in Table 2.

**TABLE 2:** The intra‐ and inter‐observer reliability for CSA

	Mean ± SD	ICC (95% CI)	*P* value
X‐ray			
Assessor 1 1.	31.7 ± 4.2		
Assessor 1 2.	32.2 ± 4.3	0.971 (0.931–0.984)	<0.001*
Assessor 2	32.0 ± 4.3	0.966 (0.953–0.975)	<0.001*
3D‐CT			
Assessor 1 1.	31.8 ± 4.4		
Assessor 1 2.	31.8 ± 4.5	0.994 (0.992–0.996)	<0.001*
Assessor 2	31.7 ± 4.6	0.987 (0.983–0.990)	<0.001*

Abbreviations: 3D‐CT, three‐dimensional computed tomography; CSA, critical shoulder angle; SD, standard deviation.

* Statistically significant.

### Correlation between Measurements from the Two Methods

**Fig. 5:**
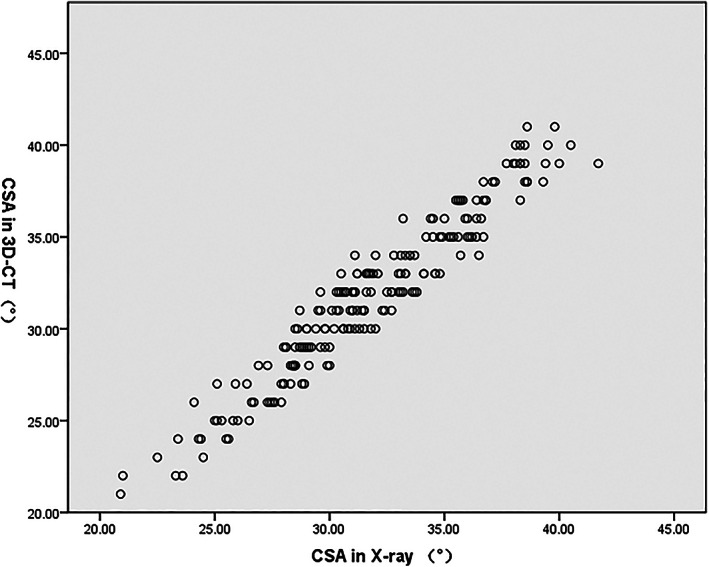
Scatter plots showing the relationship between the critical shoulder angle (CSA) measured in radiographs and 3D‐CT. Spearman's rho = 0.960.

**Fig. 6:**
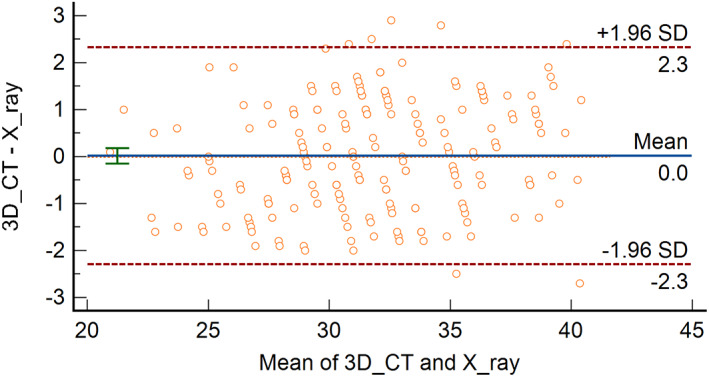
Bland–Altman plots showing the difference between critical shoulder angle measured in radiographs and 3D‐CT. SD, standard deviation.

The inter‐method correlation between the CSA measured from the standard AP radiographs and 3D‐CT was very strong (Spearman's rho = 0.960, *P* < 0.001) (Fig. 5). The mean CSA was 31.7° ± 4.2° from the standard AP radiographs and 31.8° ± 4.4° from the 3D‐CT. Wilcoxon signed‐rank test showed no significant differences between the two measurement methodologies (*P* = 0.940). Bland–Altman plots showed that the mean difference of CSA values between the standard AP radiographs and the 3D‐CT was 0.02°, and that in 95% of cases, the difference between the two methods was between −2.29° and + 2.33° (limits of agreement: −2.29° to +2.33°) (Fig. 6).

### Comparison between the Two Groups

**Fig. 7:**
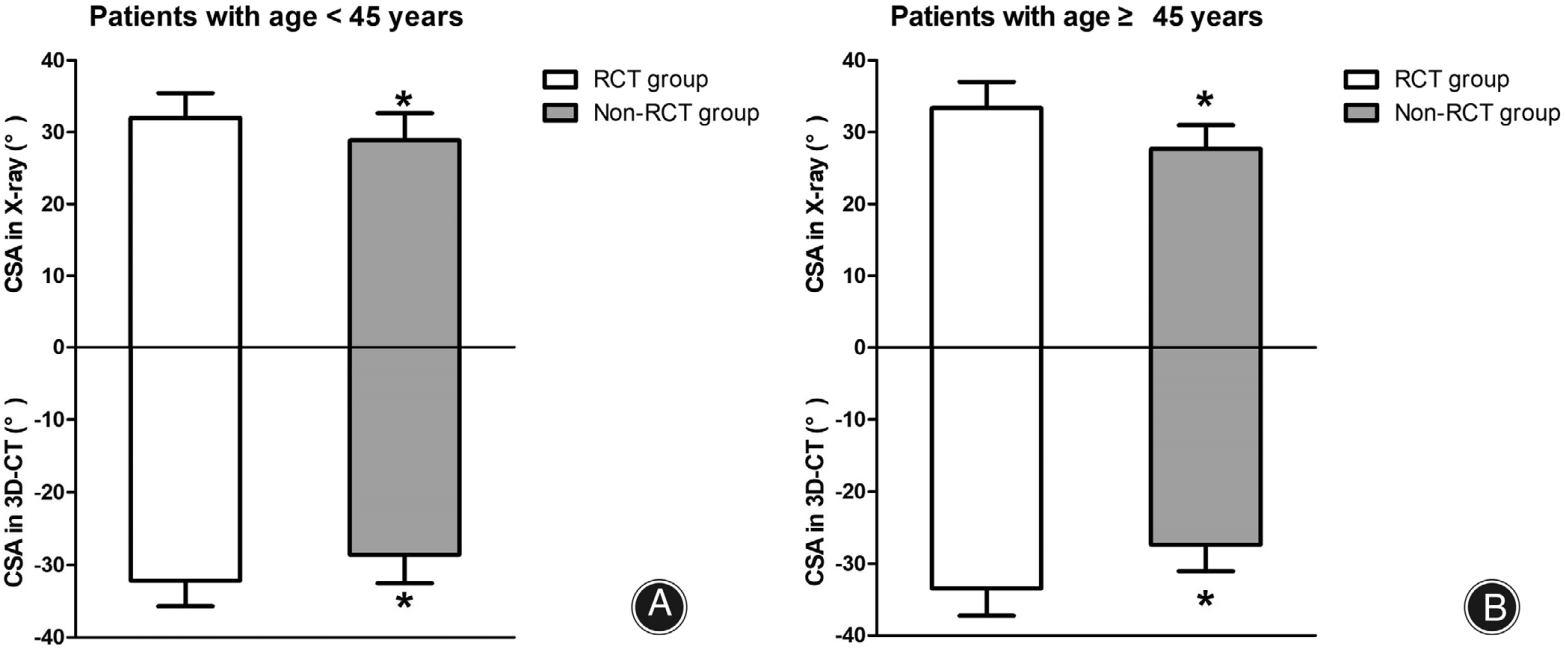
The comparison of critical shoulder angle (CSA) between patients with and without rotator cuff tears (RCTs). (A) Patients younger than 45 years. (B) Patients aged 45 years or older. The CSA values measured from radiographs are shown above the zero line, and CSA values measured from 3D‐CTs are shown below the zero line. The values are provided as the mean and standard deviation. Within a given group, significant differences (*P* < 0.05) are denoted with (*).

The CSA was significantly different between the RCTs group and non‐RCTs group (*P* < 0.001 for standard AP radiographs and 3D‐CT) (Table 1). Within the <45 years subgroup, the CSA was 32.0° ± 3.4° for the standard AP radiographs and 32.1° ± 3.6° for the 3D‐CT in the RCTs group, and 28.9° ± 3.8° for the standard AP radiographs and 28.6° ± 3.9° for the 3D‐CT in the non‐RCTs group. There were significant differences between the two groups (*P* = 0.001 for standard AP radiographs and *P* < 0.001 for 3D‐CT) (Fig. 7A). In the ≥45 years subgroup, the CSA was 33.3° ± 3.7° for the standard AP radiographs and 33.5° ± 3.8° for the 3D‐CT in the RCTs group, the CSA was 27.7° ± 3.3° for the standard AP radiographs and 27.4° ± 3.7° for the 3D‐CT in the non‐RCTs group. There were significant differences between the two groups (*P* < 0.001 for standard AP radiographs and 3D‐CT) (Fig. 7B).

### ROC Curve Analysis

**Fig. 8:**
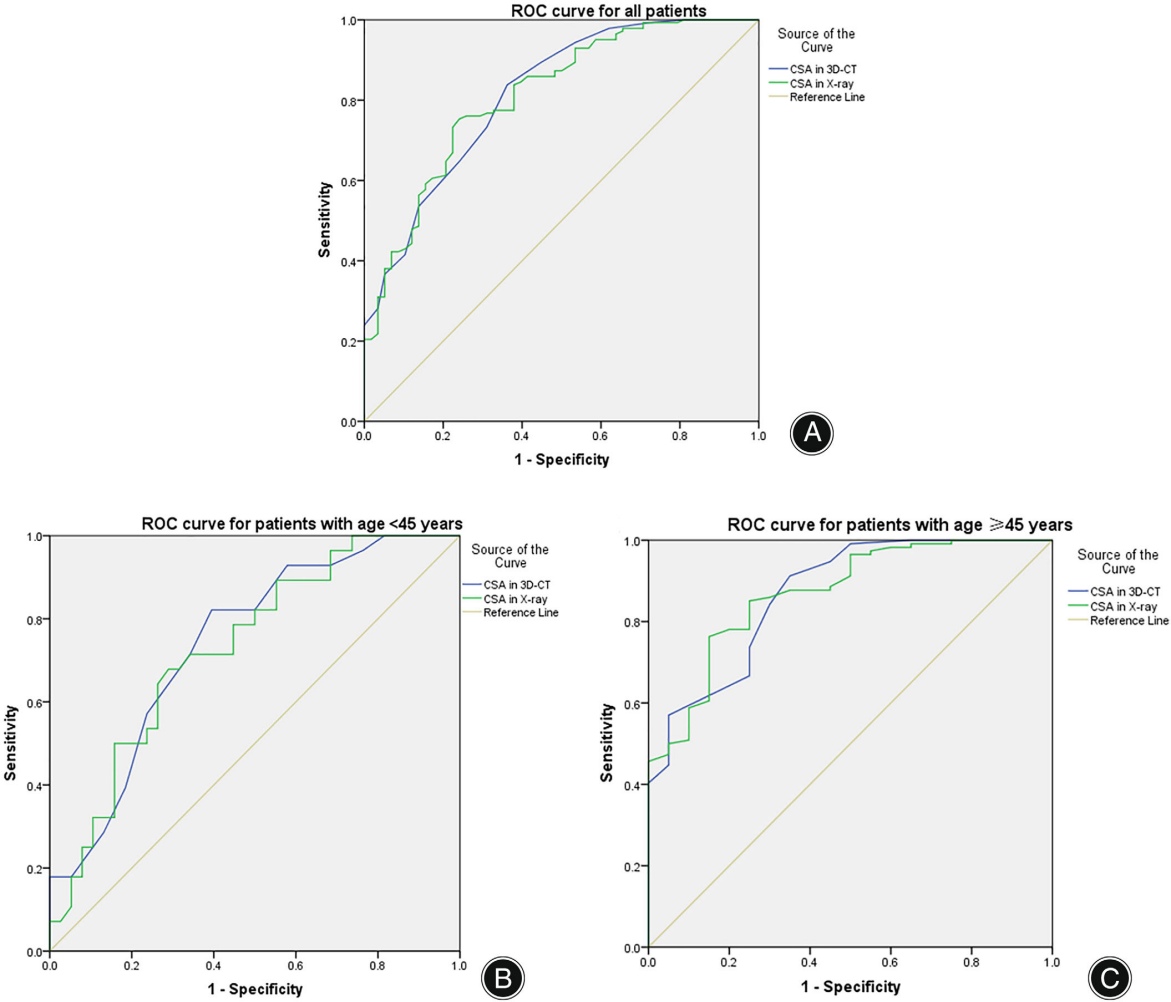
Receiver operating characteristic (ROC) curve analysis for the critical shoulder angle (CSA). (A) ROC analysis showed that for the whole cohort, the area under the curve (AUC) of the CSA was 0.812 in radiographs and 0.815 in 3D‐CT. (B) ROC analysis showed that for patients aged <45 years, the AUC of the CSA was 0.743 in radiographs and 0.731 in 3D‐CT. (C) ROC analysis showed that for patients aged ≥45 years, the AUC of the CSA was 0.869 in radiographs and 0.870 in 3D‐CT.

For the whole cohort, using ROC analysis we found that CSA measured from the standard AP radiographs (AUC = 0.812, *P* < 0.001) and the 3D‐CTs (AUC = 0.815, *P* < 0.001) showed excellent prediction for the presence of RCTs (Fig. 8A). Within the <45 years subgroup, using ROC analysis we found that CSA measured from the standard AP radiographs (AUC = 0.743, *P* = 0.001) and the 3D‐CTs (AUC = 0.731, *P* = 0.001) showed good prediction for the presence of RCTs (Fig. 8B). Within the ≥45 years subgroup, using ROC analysis we found that CSA measured in the standard AP radiographs (AUC = 0.869, *P* < 0.001) and the 3D‐CT (AUC = 0.870, *P* < 0.001) showed excellent prediction for the presence of RCTs (Fig. 8C).

## Discussion

The most important finding of this study is the high correlation between 3D‐CT and standard AP radiograph‐based CSA measurements. This suggests that 3D‐CT can be used to accurately measure the CSA. Furthermore, we confirmed an association between the measured CSA and the presence of RCTs, especially in patients aged ≥45 years.

### Accuracy and Reliability of CSA Measurement

Most shoulder AP radiographs performed in hospitals do not meet the standards set by the SH classification.^14^ Chalmers *et al*. analyzed 1433 such shoulder AP radiographs and found that up to 76% (1099 cases) did not meet the requirements.^14^, ^16^ Similarly, Tang *et al*. measured 174 AP radiographs, and only 27% (47 cases) were found to meet the standard AP radiograph criteria.^5^ The malposition of the scapula on radiographs makes it particularly difficult to obtain a high‐quality standard AP radiograph in the clinical setting; however, proper positioning is a prerequisite for accurate CSA measurements. Although, in theory, the quality of AP radiographs could be improved by systematic and normative radiologist training, non‐standard AP radiographs cannot be completely avoided. In this study, after strict radiologist training, 12.7% (46/361) cases still failed to meet the standard AP radiograph requirements, suggesting the importance of exploring alternative methods for accurately determining the CSA.

Spiegl *et al*. analyzed the correlation between MRI and standard AP radiographs to measure the CSA,^29^ finding that the MRI CSA value was significantly lower than on standard AP radiographs (28.7° ± 2.2° vs. 31.3° ± 4.4°, *P* = 0.01); the inter‐observer (ICC = 0.62) and intra‐observer (ICC = 0.68) agreements measured from MRI were lower, suggesting that MRI is not amenable to accurate measurements of the CSA.

In this study, we found that 3D‐CT and the standard AP radiographs delivered almost identical CSAs (31.8° ± 4.4° vs. 31.7° ± 4.2°, *P* = 0.940), and thus there was a strong correlation between the two methods (*r* = 0.960; *P* < 0.001). Inter‐observer (ICC = 0.987) and intra‐observer (ICC = 0.994) agreements in the 3D‐CT were perfect. Hence, we suggest that 3D‐CT is an accurate and reliable method for measuring the CSA. A similar conclusion was reached by Mah *et al*. after comparing 20 pairs of 3D‐CT and standard AP radiographs, showing that the CT‐based method is a suitable alternative for CSA measurement.^15^ Contrary to their methodology,^15^ we established a 3D coordinate system according to the scapular plane (three anatomical landmarks) before measuring CSA, which standardized the position of the 3D‐CT scapula image and eliminated measurement error arising from inconsistent 3D‐CT positioning by different investigators.

### Association between CSA and RCTs

Controversy remains regarding the relationship between CSA and RCTs. Numerous studies showed that CSA offers an objective acromial parameter to predict the presence of RCTs with greater accuracy than the acromion index (AI) or other metrics.^4^, ^6^, ^7^, ^24^, ^30^, ^31^, ^32^ In addition, higher CSAs increase the rate of retears following RCR.^33^, ^34^, ^35^, ^36^, ^37^, ^38^ However, other studies have questioned the diagnostic value and reliability of the CSA.^39^, ^40^, ^41^, ^42^ One important reason fueling the debate was the inherent measurement error of the CSA reported in previous studies. Although measuring of the CSA is simple to learn, malposition of the scapula significantly alters the apparent CSA on radiological films,^1^, ^14^ biasing the measurements. We confirmed an association between the CSA and RCTs when measured from standard AP radiographs and 3D‐CTs. Another promising finding was that CSA performed very well in diagnosing RCTs in patients aged ≥45 years. Thus, our data suggest that CSA is likely to benefit the diagnostic evaluation of patients with shoulder pain and can help predict pathology, especially in older patients.

### Strengths and Limitations

To the best of our knowledge, this study was first to investigate the consistency of CSA measurements by utilizing relatively large sample size of shoulder CT scans and matched standard AP radiographs (type A1 or C1), we believe that the findings of this study are an important prerequisite for future pre‐operative planning of acromioplasty. Despite the relative strengths of this study, certain limitations must be acknowledged. First, this was a retrospective study. Second, there may be some selection bias since some patients with poor‐quality CT scans or non‐standard AP radiographs were excluded from analysis. Lastly, patients in the control group (non‐RCTs group) had shoulder dislocations, SLAP lesions, etc. We are still unable to confirm whether those pathological conditions affect the CSA value, however, Patzer *et al*. documented that isolated SLAP lesions are associated with a low CSA.^43^

### Conclusions

CSAs measured from standard AP radiographs and 3D‐CTs are almost identical. CSA can be accurately and reliably measured from a 3D‐CT, which offers marked advantages for acromioplasty pre‐operative planning. Furthermore, CSA measured from standard AP radiographs and 3D‐CTs showed predictive value for RCTs, especially in patients aged ≥45 years, confirming an association between the CSA and RCTs.

## Author Contributions

Rui Yang conceived the study. Xiaoding Xu designed the study. Yi Long, Huijun Hu and Chuanhai Zhou performed the data analysis and manuscript preparation. Jingyi Hou created the graphs. Zhiling Wang performed the data collating. Min Zhou contributed to data collecting and analysis. Dedong Cui helped to draft the manuscript.
